# Unusual onset of a case of chronic recurrent multifocal osteomyelitis

**DOI:** 10.1186/s12969-015-0058-0

**Published:** 2015-12-12

**Authors:** M. Barrani, F. Massei, M. Scaglione, A. Paolicchi, S. Vitali, E. M. Ciancia, S. Crovella, M. C. Caparello, R. Consolini

**Affiliations:** Department of Pediatrics, Immunology and Rheumatology Section, University of Pisa, Pisa, Italy; Department of Orthopedics, University of Pisa, Pisa, Italy; Department of Diagnostic Radiology, University of Pisa, Pisa, Italy; Department of Pathology, University of Pisa, Pisa, Italy; Department of Genetics, University of Trieste, Trieste, Italy

**Keywords:** Chronic recurrent multifocal osteomyelitis, CRMO, Acute painful headache

## Abstract

**Background:**

Chronic recurrent multifocal osteomyelitis (CRMO) is a rare condition that commonly affects the clavicle and pelvis.

**Case presentation:**

We report here a case a 12 years old girl with CRMO arising with recurrent episodes of left supraorbital headache, followed by the appearance of a periorbital dyschromia. Magnetic resonance imaging (MRI) of the skull and orbits revealed an important subacute inflammatory process. Few months after, the child presented a painful swelling of the left clavicle; the histological examination of the related biopsy allowed to establish the diagnosis of CRMO.

**Conclusion:**

CRMO presenting as acute headache involving neurocranium is rare; to our knowledge this is the first recognized case in the world literature.

This pathological condition is frequently misdiagnosed as infection or neoplasm and needs a deep investigation for the differential diagnosis. The physical, laboratoristic and instrumental diagnostic investigations of the patient and the treatment employed are described in detail.

## Background

Chronic recurrent multifocal osteomyelitis (CRMO), also known as chronic nonbacterial osteomyelitis, is a rare, noninfectious inflammatory disorder that causes multifocal lytic bone lesions with swelling and pain [1]. The course of the disease is characterized by periodic exacerbations and remissions, but long term outcome remains unclear [1].

CRMO was first described in 1972 by Giedon et al. [2]; since 1972 more than 260 cases have been reported in the medical literature [3]. The prevalence of this condition is estimated at 1-2/10^6^ (Orphanet.net), but it might be underrated [4]. CRMO primarily affects young girls with a female: male ratio of 4:1 and the mean age onset is 10 years [3, 5]; the mean time from the symptoms onset to the diagnosis is 18 months, ranging from few weeks to few years [5].

CRMO may manifest only as multifocal bone lesions, but it is frequently associated with other inflammatory conditions, including peripheral arthritis, sacroileitis, psoriasis, pustulosis palmaris et plantaris, pyoderma gangrenosum [6, 7], inflammatory bowel disease (IBD) [6], severe acne, Sweet syndrome [8], Wegener’s granulomatosis [9] and Takayasu’s arteritis [7]. Individual bone lesions may be asymptomatic, but typically cause swelling, warmth, and often a dramatic degree of pain that interfere with physical function, psychosocial health, finally impairing the quality of life of the patient.

CRMO, widely believed to represent the pediatric equivalent of Synovitis, Acne, Pustulosis, Hyperostosis, Osteitis (SAPHO) syndrome, originally described in 1987 by Chamot et al. [10], is characterized by the association of osteoarticular disorders and skin manifestations.

Initially CRMO was thought to share similarities with the spondylarthropathies [11], based on the links between CRMO and SAPHO syndrome and on the presence in a substantial number of CRMO patients of psoriasis or chronic inflammatory bowel disease (Crohn’s disease or ulcerative colitis) [3, 4]. However, recent genetic data from mice with Chronic Multifocal Osteomyelitis (CMO) and humans with Majeed syndrome (CRMO with dyserythropoietic anemia), suggest that CRMO may belong to the vast family of autoinflammatory diseases [4]. They assemble a group of different conditions secondary to mutations of genes coding for proteins that play a pivotal role in the regulation of the inflammatory response. The discovery of the “inflammasome” allowed to define more than 30 autoinflammatory disorders, most of which arising during childhood [12, 13]. These entities are characterized by the presence of recurrent episodes of spontaneous inflammation and by the absence of high titles of autoantibodies or autoreactive T cells. The underlying mechanism of this condition results from the involvement of the innate immune response [12].

## Case presentation

A 12-year-old girl, suffering from recurrent episodes of left supraorbital headache since the age of 11 years, presented at Department of Pediatrics (Immunology and Rheumatology’s ambulatory) of the University of Pisa. The physiological anamnesis was apparently mute. Because of the persistence of symptoms, the child performed a brain magnetic resonance imaging (MRI) which resulted negative.

**Fig. 1:**
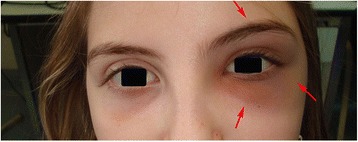
Marked hyperemia with periorbital edema of the left upper and lower eyelids

**Fig. 2:**
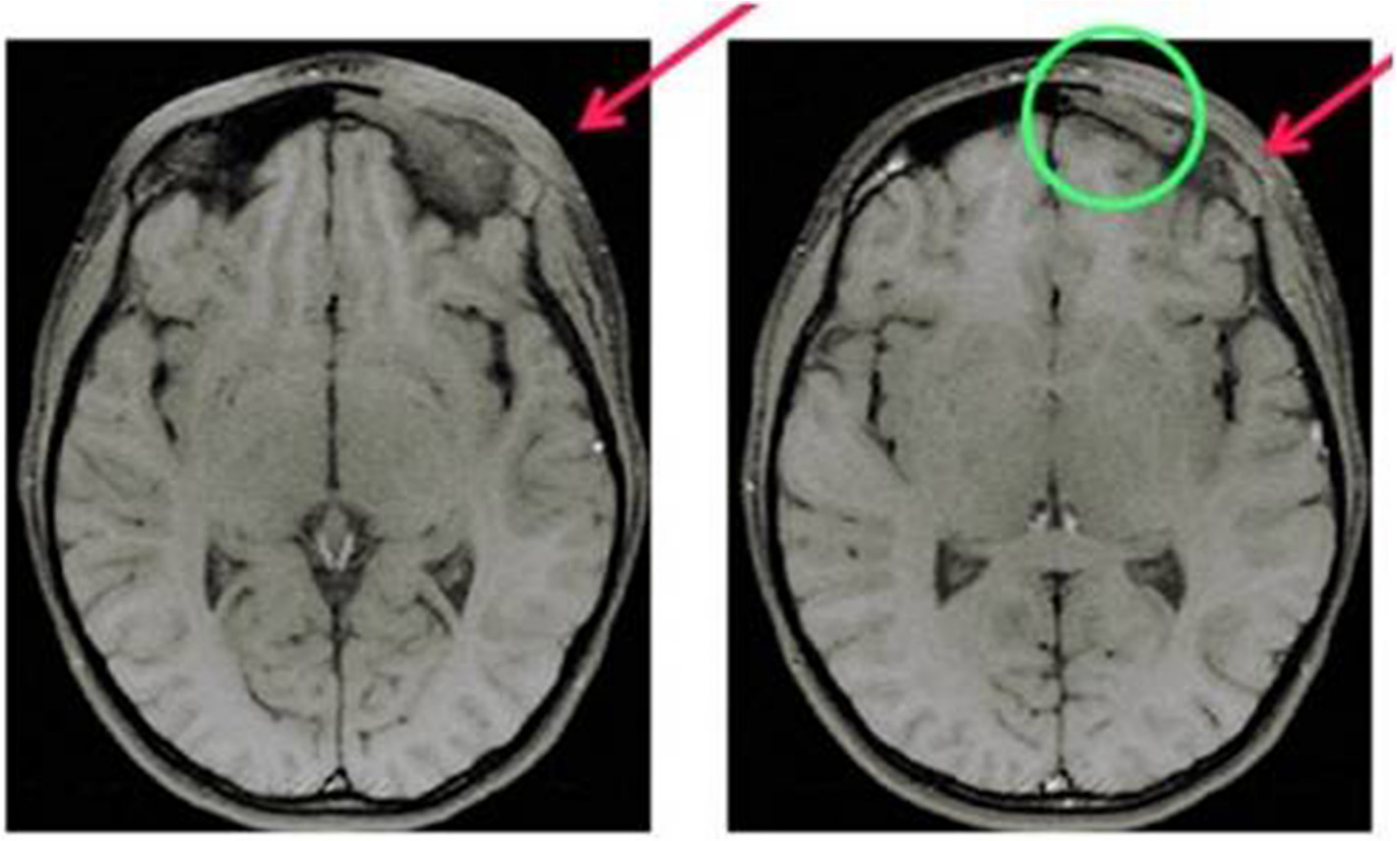
MRI: subacute inflammatory process at the level of the left orbital region with involvement of periorbital soft tissue and the bone structures

After consultation with a neuropsychiatric she started a therapy with sertraline. A month later, she presented an hyperemic periorbital circle around the left eye (Fig. 1). Due to persistence of these symptoms, a MRI of the skull and orbits was performed, which revealed an important subacute inflammatory process of the bone structures such as squama of the frontal bone, orbital process of the zygomatic bone and of the greater wing of the sphenoid bone (Fig. 2).

**Fig. 3:**
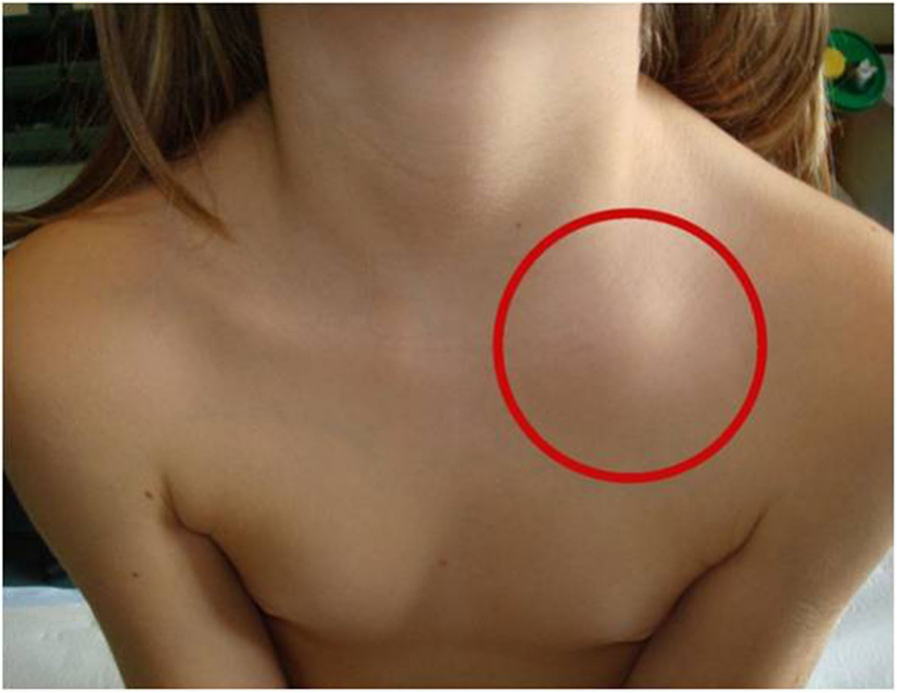
Painful swelling covered by normal skin of the sternal end of the left clavicle

After few months the child developed a painful swelling of the sternal end of the left clavicle, covered with normal skin (Fig. 3).

**Fig. 4:**
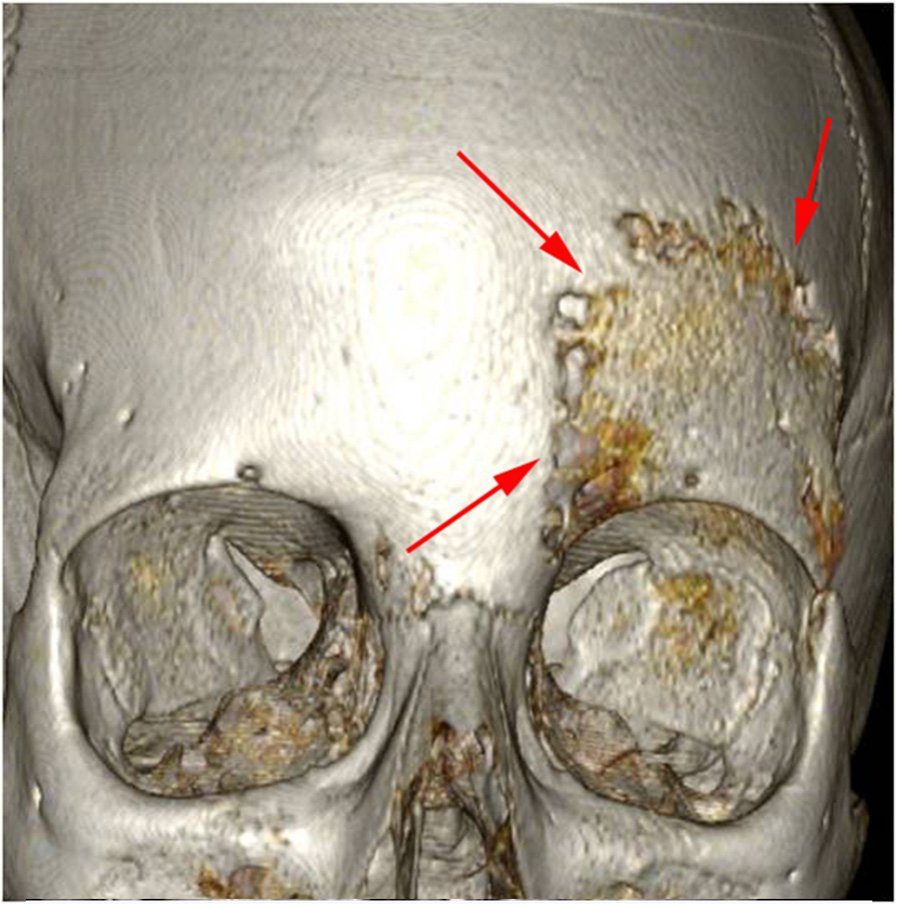
CT scan three-dimensional reconstruction

**Fig. 5:**
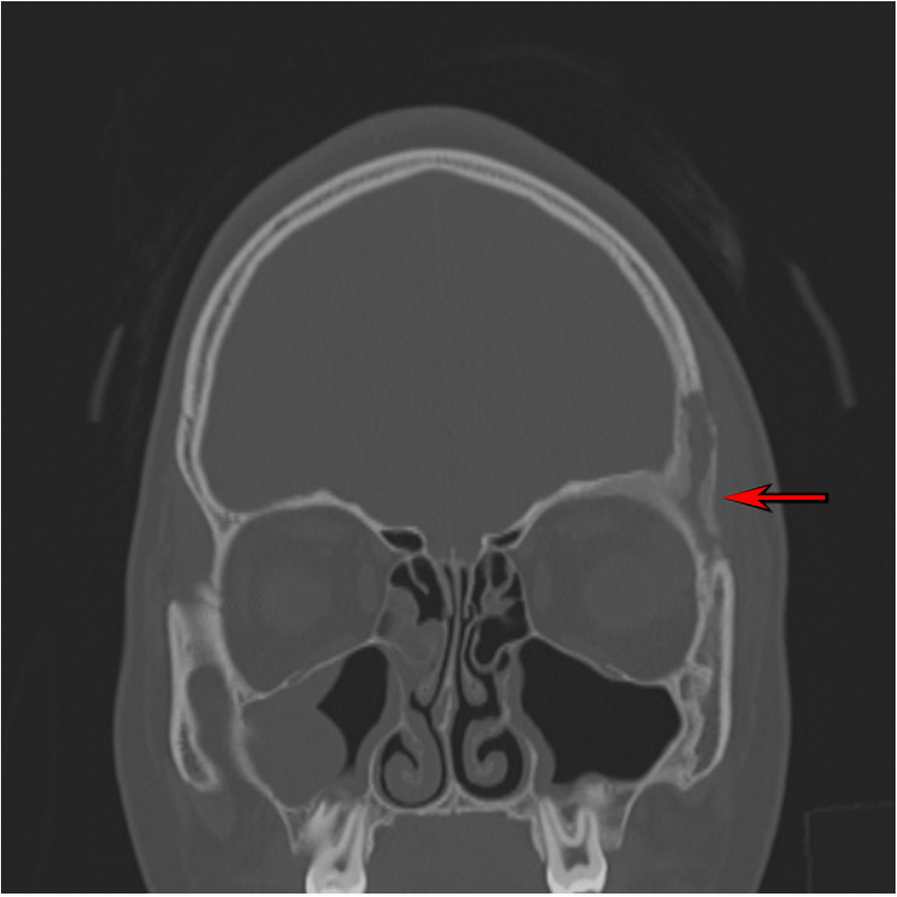
CT: remodeling and bone erosion. Note the thickness of the soft tissues (edema, inflammation)

Laboratory results showed a modest increase of inflammation parameters (ESR 49 mm/h, CRP 3.88 mg/dL) with normal complete blood count, liver and renal function. The abdominal ultrasound and the chest X-ray were normal. A whole-body scintigraphy with ^99^Tc revealed the presence of “foci” of pathological high-uptake (left frontal supraorbital area with extension to ethmoid bone and sternal end of the left clavicle), without other pathological areas in the remaining skeletal segments. X-ray of the left clavicle confirmed the presence of an area of osteolysis. Computerized Tomography Scan (CT) (Figs. 4, 5) revealed a rearrangement and thickening of the orbital spongy part of the left frontal bone and an infiltration of pathological tissue extended to the adjacent frontal squama, causing areas of osteolysis: these images were suggestive of an inflammation-infectious process.

**Fig. 6:**
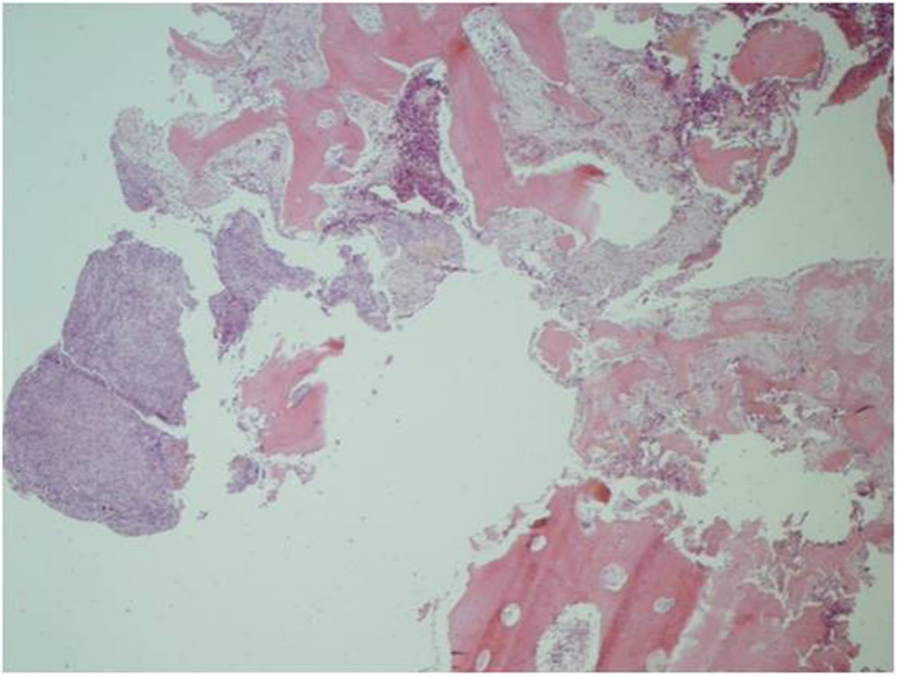
Bone with marked signs of widespread osteoblastic/osteoclastic remodeling

A biopsy of the osteolytic lesion of the left clavicle was performed: all cultures were negative; the related histopathology showed a bone with marked signs of widespread osteoblastic/osteoclastic remodeling (Fig. 6), with inter-trabeculae areas of myofibroblasts. Bone was infiltrated by a granulomatous tissue rich of lymphocytes, neutrophils, giant cells with osteoblatic shape and histiocytes with hematic pigment inside, displaying CD68 antigen to the immunohistochemistry analysis. A fine vascular reticulum CD31+ and CD34+ in the absence of cyto-keratin positive elements was observed. No neoplastic cells were identified or cells suggesting histiocytosis of Langherans cell type. This histopathological pattern strongly suggested an inflammatory reparative process; the immunohistochemistry staining (pS-100, CD1a, actin/1A4, CD68/KP-1, Ki-67/MB-1) definitively excluded the presence of neoplastic tissue. Clinical history, physical signs, instrumental investigations and histopathological pattern were highly suggestive of CRMO. After starting anti-inflammatory therapy with naproxen, the child carried out a clinical, biological and instrumental (MRI total body with STIR sequences) follow-up every 6 months. After 4 months from diagnosis the clinical picture was negative and a MRI showed the improvement of previous findings; therefore the therapy with naproxen was discontinued. In the following 12 months the child again experienced recurrent attacks of headache associated with bone painful mainly localized to upper extremities. Although MRI STIR sequences total body were negative, the observed picture of “bone remodeling” of the cranial region, in absence of enhanced inflammatory markers, led us to plan therapy with bisphosphonates.

## Conclusion

Chronic recurrent multifocal osteomyelitis (CRMO) is a rare, noninfectious inflammatory recurrent disorder, with periodic exacerbations and remissions, but long term outcome remains unclear [1].

The diagnosis is of exclusion and it is based on the clinical and radiological data. In most cases biopsy and bone histology are mandatory to confirm the diagnosis.

CRMO may manifest as isolated multifocal bone lesions, but is frequently associated with other inflammatory conditions [6–9].

The peculiarity of our case concerns especially the initial site of disease, in the orbital region, causing recurrent headache attacks.

Therefore, the primary unusual location caused difficulties in the differential diagnosis with other pathological conditions such as: Langerhans cell histiocytosis, osteoblastoma, osteosarcoma.

The appearance of a second location of the disease at the level of the medial portion of left clavicle (considered as “typical site” in 5 % of cases reported in literature [14]), and the complexity of the investigations above described, allowed the diagnosis of CRMO.

Our patient presents some similarities with a previously described case of CRMO arising with recurrent painful swelling of cheeks [15]. In both cases a cranial involvement is reported, but with different osseous sites (the mandibular ramus in the previous one and frontal, orbital and sphenoid bones in our case), associated with slightly elevated inflammation parameters. Therefore the peculiarity of our case is the initial site of the disease in the neurocranium (the first case described), causing misleading headache attacks.

CRMO has been recently considered an inflammatory disease, as evidenced by genetic data derived from experimental murine model and from humans affected by Majeed Syndrome, showing the mutations of genes involved in the regulation of inflammatory response (PSTPIP2 and LPIN2 respectively localized on chromosome 18p) [16].

Interestingly, the observed low mannose-binding lectin (MBL) production in some CRMO patients, presenting with MBL genetic variants, suggested a linkage of CRMO with the involvement of the innate immunity [17]. The MBL is a serum protein that plays a role in the innate immune response; it binds to carbohydrates on the surface of a wide range of pathogens, where it activates the complement system or directly acts as opsonin, by enhancing phagocytosis, through the binding to cell surface receptor expressed on phagocytic cells [17]. Polymorphism in the first exon of the MBL2 gene is known to lead to a diminished production of MBL, as well as an impairment of its function. Recent studies suggested that the variations in MBL serum concentration are associated with increased risk of infectious and autoimmune diseases [17]. In our patient, MBL2 genetic screening was performed by polymerase chain reaction amplification from genomic DNA and subsequent direct sequencing of the amplicons. The analysis was performed on six functional nucleotide variations, (three in the promoter region (−550, −221,+4) and in the exon 1 (position 52,54,57) of the gene, that are in linkage disequilibrium and whose haplotypes are associated with different quantitative production of MBL. The combined genotype, in our patient, was LYQA/LYQA, associated with a normal protein production.

CRMO has been long believed to have a benign self-limited course and to resolve without leaving any consequential residual damages, but recent data suggest that physical impairments may persist in up to 50 % of patients [3]. They consist in chronic pain and bone deformities, impairing physical and emotional health [3, 5].

The treatment of CRMO has been widely empiric; although NSAIDs remain the first choice, the alternative therapy is represented by bisphosphonates and biologic drugs such as TNF antagonists (etanercept) or inhibitors of IL-1 (anakinra), but further studies are needed to evaluate the safety of long-term of these drugs in children. Furthermore, a multidisciplinary approach involving different specialties (pediatrics, rheumatology, radiology, orthopedics, pathology and in selected cases also physiotherapy, plastic surgery, gastroenterology and dermatology) is necessary.

The child was initially treated with a first-line drug, belonging to the class of anti-inflammatory drugs, which resulted to a progressive resolution of the lesions in few months.

However, the recurrence of bone pain episodes, observed during the follow-up of patient, although not associated with perceptible alterations at the instrumental exams (MRI STIR sequences), led us to introduce a second line drug, in attempt to reduce the progression of the disease. The therapeutic choice turned towards the class of bisphosphonates, according to the most recent indications of the literature [16]. Their anti-osteoclastic activity has been clearly established by the described morphological and functional cellular alterations, typical of the active phase of bone resorption such as the loss of the brush border and of the cytoplasmic vacuoles [18]; the reduction of the lactic acid production, the proton accumulation, the synthesis of enzymes lysosomes and prostaglandins, of the acid phosphatase and pyrophosphatase, fundamental to the phagocytic activity of osteoclasts has also been reported [18].

Therefore the variety of the molecular targets of bisphosphonates indicate them as a relevant therapeutic approach of cases of CRMO, characterized by the predominance of the bone involvement on the systemic inflammation [4], in order to prevent the bone damage and the severe impairment of quality of life.

## Consent

Written informed consent was obtained from the patient’s parents for publication of this case report and accompanying images. A copy of the written consent is available for review by the Editor-in-Chief of this journal.

## Abbreviations

CRMO: Chronic recurrent multifocal osteomyelitis
MRI: Magnetic resonance imaging
IBD: Inflammatory bowel disease
SAPHO: Synovitis, Acne, Pustulosis, Hyperostosis, Osteitis syndrome
TC: Computerized Tomography Scan
MBL: Low mannose-binding lectin
NSAIDs: Non steroidal anti-inflammatory drugs
